# Evaluation of Aortic Dilation in Patients With Hypertrophic Cardiomyopathy in a Tertiary Center for Cardiovascular Disease

**DOI:** 10.1155/crp/7137001

**Published:** 2026-07-05

**Authors:** Tahereh Babaei, Kosar Babaei, Golnaz Houshmand, Farzaneh Amini, Parham Rabiee, Zeinab Norouzi, Hamideh Khesali, Raheleh Kaviani

**Affiliations:** ^1^ Department of Cardiology, School of Medicine, Iran University of Medical Sciences, Tehran, Iran, iums.ac.ir; ^2^ Cardiovascular Research Center, Rajaie Cardiovascular Institute, Tehran, Iran; ^3^ Students Research Committee, Neyshabur University of Medical Sciences, Neyshabur, Iran, neyshabur.ac.ir; ^4^ Cardiovascular Imaging Research Center, Rajaie Cardiovascular Institute, Tehran, Iran; ^5^ Department of Biostatistics and Epidemiology, Student Research Committee, Faculty of Health, Mazandaran University of Medical Sciences, Sari, Iran, mazums.ac.ir; ^6^ Echocardiography Research Center, Rajaie Cardiovascular Institute, Tehran, Iran

**Keywords:** aortic dilation, echocardiography, hypertrophic cardiomyopathy, sinus of Valsalva dilation

## Abstract

**Background:**

Hypertrophic cardiomyopathy (HCM) is a common genetic cardiac disease primarily affecting the left ventricle. While the disease is traditionally associated with myocardial hypertrophy and left ventricular outflow tract (LVOT) obstruction, aortic dilation has emerged as a potentially serious but underrecognized complication. This study aimed to determine the prevalence of aortic dilation in HCM patients and to identify associated clinical and echocardiographic factors.

**Methods:**

In this cross‐sectional, retrospective study, 216 adult patients diagnosed with HCM at Rajaie Cardiovascular Institute (Tehran, Iran) from 2016 to 2021 were evaluated. Patients with significant valvular disease, congenital aortic anomalies, prior aortic surgery, or other cardiomyopathies were excluded. Clinical data and echocardiographic measurements, including aortic dimensions indexed to body surface area (BSA), were collected. Cardiac magnetic resonance imaging (MRI) data of myocardial fibrosis were also assessed in a subset of patients. Logistic regression was used to identify factors independently associated with aortic dilation.

**Results:**

Dilated ascending aorta was present in 56 patients (26.3%), while 12 patients (5.6%) had dilation of the sinus of Valsalva (SOV). Dilated ascending aorta was significantly associated with older age (*p* = 0.001), hypertension (*p* = 0.001), and increased BSA (*p* = 0.002). LV end‐systolic diameter (LVESD) also showed a significant correlation (*p* = 0.002). Multivariate analysis identified age (OR = 1.063), hypertension (OR = 5.49), LVESD (OR = 3.25), and right ventricular hypertrophy (OR = 4.99) as independent predictors of ascending aorta dilation. SOV dilation was similarly associated with age (OR = 1.117) and lower weight (OR = 0.915). Although myocardial fibrosis was more common in patients with ascending aorta dilation, this did not reach statistical significance (*p* = 0.006).

**Conclusion:**

Aortic dilation is a relatively common finding in patients with HCM and is significantly associated with age, hypertension, and structural cardiac remodeling. Routine evaluation of the ascending aorta and SOV in HCM patients may aid in the early detection and management of this potential complication.

## 1. Introduction

**FIGURE 1:**
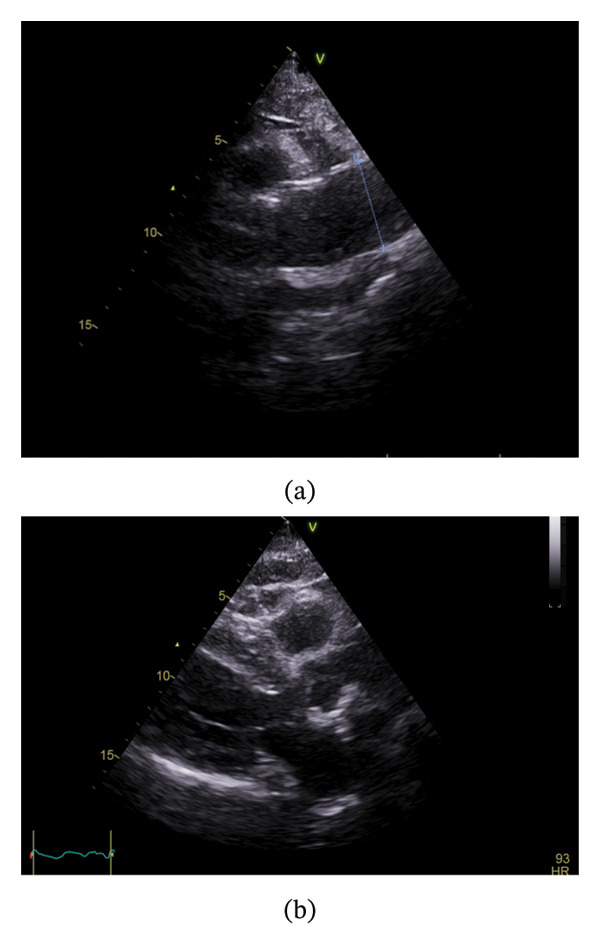
Parasternal long‐axis view in transthoracic echocardiography, (a) at the 3rd intercostal space, showed dilated ascending aorta at the mid‐level as measured 4.1 cm (shown by blue dotted line), in a patient with (b) hypertrophic cardiomyopathy (parasternal long‐axis view at 4th intercostal space).

Hypertrophic cardiomyopathy (HCM) is a prevalent hereditary cardiac disease, affecting 0.2%–0.5% of the general population. It is marked by hypertrophy of left ventricular walls, diastolic dysfunction, and, sometimes, left ventricular outflow tract (LVOT) obstruction [1, 2]. This disease may result in heart failure, ventricular arrhythmias, and even sudden cardiac death [3]. The coexistence of aortic root dilation in patients with HCM and its consequences, such as dissection, is less known. Research indicates that aortic dilation in individuals with HCM (Figure 1) may result not only from hemodynamic alterations linked to LVOT obstruction but also from structural modifications in the aorta caused by mechanical stress and genetic anomalies [4–6]. The incidence of aortic dilation varies among various patient populations with HCM, with approximately 29% exhibiting aortic dilation [7, 8]. Echocardiography early detects the aortic dilation and related conditions in these patients, providing chamber sizes, diastolic and systolic function, and aortic diameters [9, 10].

Prior research has demonstrated a notable correlation between certain clinical and demographic variables and aortic dilation in individuals with HCM [11, 12]. Despite existing evidence, the literature is limited in individuals with HCM within the Iranian community. Given that hereditary and environmental variables may significantly influence the manifestation of this disorder, it is essential to examine the prevalence and risk factors linked to aortic dilation in patients with HCM in our nation. This study aimed to ascertain the incidence of aortic dilation and identify its associated characteristics in adult patients with HCM [13], to propose efficient techniques for managing and preventing its related consequences [14, 15].

## 2. Method

This cross‐sectional, retrospective study encompassed patients referred to Rajaie Cardiovascular Institute in Tehran from 2016 to 2021, with a confirmed diagnosis of HCM. Patients with valvular aortic stenosis (AS), fixed sub valvular AS, supra valvular AS, moderate aortic insufficiency (AI) or greater, congenital structural anomalies of the aorta, cardiomyopathies other than HCM, prior history of aortic or other heart valve surgery, coronary artery stenosis (exceeding 50% in angiography), and patients with renal failure or severe systemic diseases that influence echocardiographic parameters were excluded.

This study received approval from the Research Ethics Committee of Rajaie Cardiovascular Institute, and consent was waived due to the retrospective nature of the study. Demographic data including age, gender and body surface area (BSA), heart rate (HR), rhythm, systolic blood pressure (SBP) and diastolic blood pressure (DBP), past medical history such as hypertension (HTN), diabetes mellitus (DM), nonsignificant coronary artery disease (CAD) with stenosis lower than 50%, and the presence of an implanted cardioverter defibrillator (ICD) were collected from patients’ electronic medical records.

Echocardiographic data including aortic dimensions, left ventricular end‐diastolic volume index (LVEDVi), left ventricular end‐diastolic diameter (LVEDD), left ventricular end‐systolic diameter (LVESD), left ventricular ejection fraction (LVEF), maximum left ventricle (LV) wall thickness, septal e’ velocity, average ratio of E velocity in mitral inflow to septal e’ velocity and ratio of E velocity in mitral inflow to lateral e’ velocity, left atrial volume index (LAVi), presence of systolic anterior motion (SAM) of anterior mitral valve leaflet (AMVL), presence of LVOT obstruction both at rest and following the Valsalva maneuver, presence and grade of mitral regurgitation (MR) and AI, presence of right ventricular hypertrophy (RVH), systolic pulmonary artery pressure (sPAP), and presence and degree of pulmonary hypertension (PH) were collected from echocardiography reports of patients that all were done by Level 3 competency echocardiography and based on current approved guidelines [16].

Aortic dimensions, including sinus of Valsalva (SOV) and ascending aorta size, were assessed in the parasternal long‐axis view at end‐diastole using leading edge to leading edge technique at the SOV (sinus‐to‐sinus) and at the site of the maximum ascending aortic diameter. Aortic dilation was assessed using sex‐ and BSA‐indexed reference values: The SOV normal range was considered 1.7 ± 0.2 cm/m^2^ in men and 1.8 ± 0.2 cm/m^2^ in women. Similarly, the proximal ascending aorta normal range was considered 1.5 ± 0.2 cm/m^2^ in men and 1.6 ± 0.3 cm/m^2^ in women [16].

LV systolic dysfunction was defined as LVEF < 60%. LV enlargement was defined as LVEDVi > 74 cc/m^2^ in men and > 61 cc/m^2^ in women. LA enlargement was defined as LAVi > 34 cc/m^2^. PH was defined as sPAP > 35 mmHg on echocardiography. AI and MR were graded as mild, moderate, or severe based on current ACC/AHA guidelines.

**FIGURE 2:**
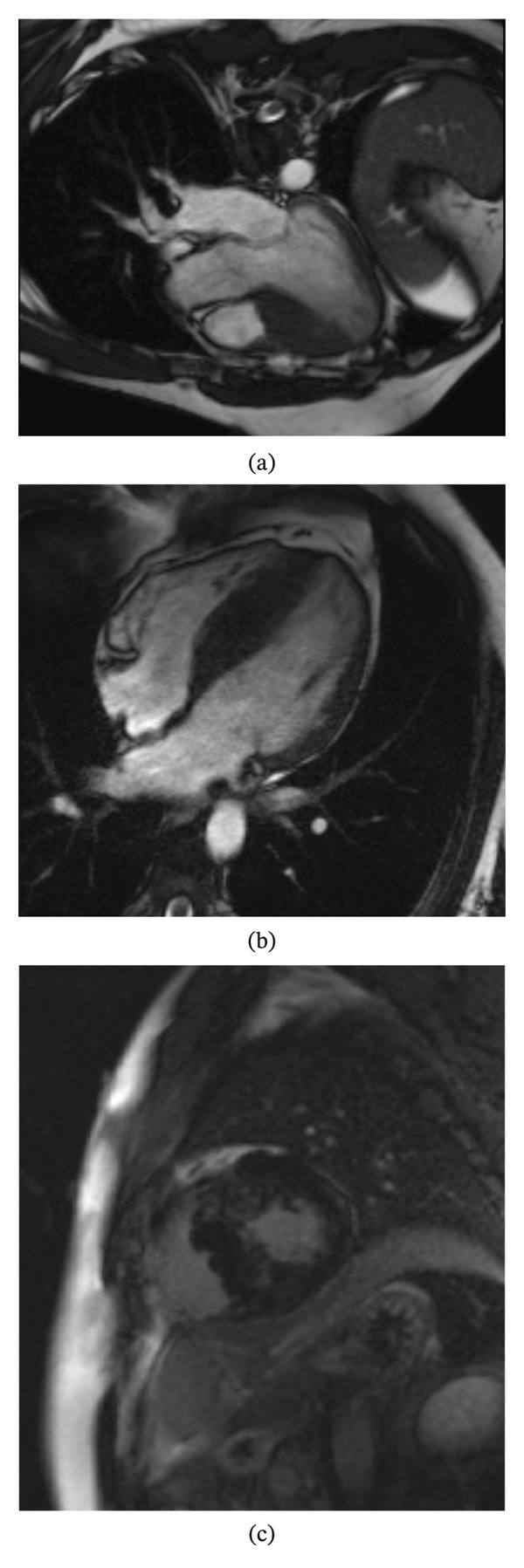
(a‐b) Three‐chamber and four‐chamber SSFP images showing asymmetric hypertrophy of the basal to apical septal wall. (c) Mid‐level short‐axis late gadolinium enhancement (LGE) image showing patchy mid‐wall fibrosis in the hypertrophied septum.

The patients who underwent cardiac magnetic resonance imaging (MRI) were evaluated for the presence of myocardial fibrosis, and the data were collected from their reports (Figure 2).

All the mentioned data were collected from documents at the time of the initial comprehensive assessment at our center.

### 2.1. Statistical Analysis

Statistical analyses were conducted in two sections: descriptive and analytical. In the descriptive section, qualitative variables were reported based on count and percentage, while quantitative variables were described using mean and standard deviation. In the analytical section, normality was assessed using the Kolmogorov–Smirnov test and the Shapiro–Wilk test, with a significance level of 0.05. Parametric tests were applied to variables with a normal distribution. The Student’s *t*‐test was used to compare quantitative variables between two groups, and Fisher’s exact test was employed for comparing qualitative variables. We evaluate the relationship between all independent variables and the response variables using the logistic regression model. Prior to analysis, we performed dimensionality reduction due to overdispersion, high dimensionality, and multicollinearity among the independent variables. Only the most important variables, which showed no multicollinearity, were included in the model. Next, we tested the conditions and criteria that evaluate the adequacy of the model fit, concluding that the logistic regression model is appropriate. The results of the Hosmer and Lemeshow Test indicated a *p* value greater than 0.05, with values reported as 0.415 and 0.237 for the variables dilated ascending aorta and dilated SOV, respectively. The Cox & Snell R Square and Nagelkerke R Square values indicate the proportion of variance in the dependent variable explained by the model, showing that 30%–40% and 39%–42% of the variability in dilated ascending aorta and dilated SOV, respectively, can be accounted for by these variables. Finally, the overall percentage indicates that the model can adequately explain the dependent variable. Currently, the model classifies 94% and 79% of the total cases correctly for the variables dilated ascending aorta and dilated SOV, respectively.

Finally, all analyses were performed using SPSS 26 at a significance level of 0.05.

## 3. Results

Considering the exclusive criteria, 216 patients were enrolled in our retrospective cross‐sectional study. The mean age was 48.3 ± 15.2 years, and 58.8% (*n* = 127) were male. Table 1 shows patients’ characteristics and past medical history. The patients were divided into two groups with and without aortic dilation, including ascending aorta and/or SOV dilation [16]. Patient’s characteristics, past medical history, and echocardiographic data were compared between 2 groups. Tables 2 and 3 show the comparison of patients with and without dilated ascending aorta and dilated SOV, respectively. Among 216 patients, 87 underwent cardiac MRI. Myocardial fibrosis in cardiac MRI was compared between the two groups, too.

**TABLE 1:** Patient’s characteristics, past medical history, and echocardiographic data.

**Variable**	**Mean ± SD or frequency**		

Age (years)	48.3 ± 15.2		
Male (%)	127 (58.8%)		
Diabetes (%)	6.5		
HTN (%)	20.8		
ICD (%)	31.9		
AF (%)	19.9		
BSA (m^2^)	1.82 ± 0.2		
Heart rate (beat per minute)	71.2 ± 6.9		
SBP (mmHg)	118.2 ± 15.6		
DBP (mmHg)	73.8 ± 9.4		

**Kind of HCM**	**Numbers (frequency %)**		

Obstructive	82 (38%)		
Nonobstructive	134 (62%)		

**Distribution of aortic dilation frequency in the study population**	***n* (%)**	**Mean ± SD**	

Dilated ascending aorta	66 (30.6%)	3.6 ± 0.3 cm	
Dilated ascending aorta/BSA	56 (26.3%)	2.06 ± 0.3 cm/m^2^	
Dilated sinus of Valsalva	19 (8.8%)	3.7 ± 0.2 cm	
Dilated sinus of Valsalva/BSA	12 (5.6%)	2.2 ± 0.2 cm/m^2^	

**Echocardiographic quantitative parameters**	**Minimum**	**Maximum**	**Mean ± SD**

LVEDVi (cc/m^2^)	18.0	152.0	47.5 ± 14.4
LVEDD (cm)	2.50	6.8	4.3 ± 0.6
LVEF (%)	20	70	52.74 ± 8.25
LVESD (cm)	0.60	6.0	2.8 ± 0.7
LAVi (cc/m^2^)	13.0	254.0	43.1 ± 23.4
Maximum LV wall thickness (cm)	1.10	3.9	2.1 ± 0.5
Rest LVOT gradient (mmHg)	30.0	140.0	61.7 ± 28.8
Provoked LVOT gradient (mmHg)	30.0	136.0	69.8 ± 27.8
Average E/e’	1.5	36	11.5 ± 5.9
Septal E/e’	2	45	14.2 ± 6.6
Systolic PAP (mmHg)	14.0	68.0	34.1 ± 10.3

**Echocardiographic qualification parameters**	**Yes/no**	**Frequency (%)**	

LV systolic dysfunction	No	168 (77.8%)	
	Yes	48 (22.2%)	

LV enlargement	No	201 (93.0%)	
	Yes	15 (7%)	

LA enlargement	No	72 (33.3%)	
	Yes	144 (66.7%)	

SAM of AMVL	No	92 (42.6%)	
	Yes	124 (57.4%)	

PH	No	142 (65.7%)	
	Yes	74 (34.3%)	

MR	No	2 (0.9%)	
	Yes	99.1	

AI	No	117 (54.1%)	
	Yes	99 (45.9%)	

*Note:* HCM: hypertrophic cardiomyopathy, LVEDVi: left ventricular end‐diastolic volume index, LVEDD: left ventricular end‐diastolic diameter, HTN: hypertension.

Abbreviations: AF, atrial fibrillation; AI, aortic insufficiency; AMVL, anterior mitral valve leaflet; BSA, body surface area; DBP, diastolic blood pressure; ICD, implanted cardioverter defibrillator; LAVi, left atrium volume index; LV, left ventricle; LVEF, left ventricle ejection fraction; LVESD, left ventricular end‐systolic diameter; LVOT, left ventricular outflow tract; MR, mitral regurgitation; PAP, pulmonary arterial pressure; PH, pulmonary hypertension; SAM, systolic anterior motion; SBP, systolic blood pressure.

**TABLE 2:** Relationship between dilated ascending aorta and studied variables.

**Group variable**		**Dilated ascending aorta**		**p** **value**
		**No (*n* = 150)**	**Yes (*n* = 66)**	

**Characteristics**		**Mean ± SD**		

Age (years)		45.37 ± 14.65	56.80 ± 13.93	0.001^∗^
Height (cm)		168.4 ± 8.96	164.2 ± 10.69	0.004^∗^
Weight (kilograms)		76.90 ± 14.09	70.57 ± 13.32	0.004^∗^
BSA (m^2^)		1.84 ± 0.205	1.74 ± 0.201	0.002^∗^
Heart rate (beat/minute)		71.29 ± 7.60	70.94 ± 4.52	0.748
SBP (mmHg)		117.4 ± 15.32	120.5 ± 16.30	0.197
DBP (mmHg)		73.49 ± 9.23	74.92 ± 9.89	0.326

		**Number (%)**		

Sex	Female	67 (42%)	22 (38.6%)	0.641
	male	92 (57.9%)	35 (61.4%)	

HTN	No	134 (85.4%)	34 (60.7%)	0.001^∗^
	yes	23 (14.6%)	22 (39.3%)	

DM	No	146 (93%)	54 (94.7%)	0.764
	yes	11 (7.1%)	3 (5.4%)	

ICD	No	108 (68.4%)	38 (66.7%)	0.815
	yes	50 (31.6%)	19 (33.3%)	

AF	No	128 (81.5%)	43 (75%)	0.326
	yes	29 (18.5%)	14 (24.6%)	

*MRI data*				
Myocardial fibrosis	No	22 (32.4%)	2 (10.5%)	0.06
	yes	46 (67.6%)	17 (89.5%)	

*Echocardiographic data*				
LVEDVi (cc/m^2^)		47.12 ± 12.79	48.69 ± 20.92	0.518
LVEDD (cm)		4.30 ± 0.64	4.41 ± 0.71	0.325
LVESD (cm)		2.80 ± 0.64	3.14 ± 0.84	0.002^∗^
LAVI (cc/m^2^)		43.95 ± 26.15	40.97 ± 14.34	0.437
Maximum LV wall thickness (cm)		2.17 ± 0.54	2.14 ± 0.53	0.762
Rest LVOT gradient (mmHg)		65 ± 30.43	53.5 ± 23.06	0.154
Provoked LVOT gradient (mmHg)		68.48 ± 26.16	72.76 ± 33.99	0.654
Average E/e′		11.64 ± 6.02	11.30 ± 5.75	0.712
Septal E/e′		14.18 ± 6.34	14.24 ± 7.46	0.950
Systolic PAP (mmHg)		33.72 ± 10.16	35.53 ± 11.03	0.287
LVEF (%)	> 50	119 (74.8%)	39 (69.6%)	0.448
	=< 50	40 (25.2%)	17 (30.4%)	

LV enlargement	No	149 (93.7%)	52 (91.2%)	0.548
	yes	10 (6.3%)	5 (8.8%)	

LA enlargement	no	52 (32.7%)	19 (33.3%)	0.931
	yes	107 (67.3%)	38 (66.7%)	

PH	no	105 (66%)	37 (64.9%)	0.872
	yes	54 (34%)	20 (35%)	

Kind of HCM	obstructive	60 (37.7%)	22 (38.6%)	0.909
	Nonobstructive	99 (62.3%)	35 (61.4%)	

RVH	No	149 (93.7%)	49 (86%)	0.092
	Yes	10 (6.3%)	8 (14%)	

SAM of AMVL	No	64 (40.3%)	28 (49.1%)	0.245
	Yes	95 (59.7%)	29 (50.9%)	

Significant LVOT gradient at rest	No	114 (71.7%)	39 (68.4%)	0.640
	Yes	45 (28.3%)	18 (31.6%)	

Significant provoked LVOT gradient	No	85 (75.2%)	31 (70.5%)	0.563
	Yes	28 (24.8%)	13 (27.5%)	

*Note:* LVEDVI: left ventricular diastolic volume index, LVEDD: left ventricular diastolic diameter, HTN: hypertension, HCM: hypertrophic cardiomyopathy.

Abbreviations: AF, atrial fibrillation; AI, aortic insufficiency; AMVL, anterior mitral valve leaflet; BSA, body surface area; DBP, diastolic blood pressure; DM, diabetes mellitus; ICD, implanted cardioverter defibrillator; LA, left atrium; LAVI, left atrium volume index; LV, left ventricle; LVEF, left ventricle ejection fraction; LVESD, left ventricular end‐systolic diameter; LVOT, left ventricular outflow tract; MR, mitral regurgitation; PAP, pulmonary arterial pressure; PH, pulmonary hypertension; RVH, right ventricle hypertrophy; SAM, systolic anterior motion; SBP, systolic blood pressure.

^∗^ *p* value < 0.05.

**TABLE 3:** Relationship between dilated sinus of valsalva and studied variables.

**Group variable**		**Dilated SOV**		**p** **value**
		**No (*n* = 204)**	**Yes (*n* = 12)**	
		**Mean ± SD**		

*Characteristic*				
Age (years)		47.49 ± 14.79	63.08 ± 16.68	0.001^∗^
Height (cm)		167.7 ± 9.30	160.33 ± 12.36	0.009^∗^
Weight (kilograms)		75.84 ± 13.98	64.75 ± 13.10	0.008^∗^
BSA (m^2^)		1.83 ± 0.205	1.65 ± 0.21	0.004^∗^
Heart rate (beat/minute)		70.94 ± 5.36	75.41 ± 19.36	0.442
SBP (mmHg)		118.1 ± 15.48	120.3 ± 18.26	0.635
DBP (mmHg)		73.73 ± 9.14	76.54 ± 13.82	0.336

		**Number (%)**		

Sex	Female	84 (41.2%)	5 (41.7%)	0.973
	male	120 (58.8%)	7 (58.3%)	

HTN	No	163 (80.9%)	5 (45.5%)	0.013^∗^
	Yes	39 (19.3%)	6 (54.5%)	

DM	No	188 (93%)	12 (100%)	0.614
	Yes	14 (7%)	0	

ICD	No	139 (68.5%)	7 (58.3%)	0.528
	Yes	64 (31.5%)	5 (41.7%)	

AF	No	160 (79.8%)	11 (91.7%)	0.467
	Yes	42 (20.8%)	1 (8.3%)	

*MRI data*				
Myocardial fibrosis	No	24 (29.3%)	0 (0%)	0.316
	Yes	58 (70.7%)	5 (100%)	

*Echocardiographic data*				
LVEDVi (cc/m^2^)		47.17 ± 13.88	53.75 ± 31.47	0.486
LVEDD (cm)		4.32 ± 0.64	4.45 ± 1.04	0.513
LVESD (cm)		2.87 ± 0.69	3.15 ± 1.09	0.203
LAVi (cc/m^2^)		43.50 ± 23.91	37.09 ± 14.07	0.381
Maximum LV wall thickness (cm)		2.16 ± 0.53	2.29 ± 0.67	0.412
Rest LVOT gradient (mmHg)		62.25 ± 28.73	28 ± 13.8	0.232
Provoked LVOT gradient (mmHg)		70.52 ± 27.95	57.50 ± 31.81	0.526
Average E/e’		11.56 ± 5.94	11.39 ± 5.97	0.923
Septal E/e’		14.20 ± 6.66	14.13 ± 6.81	0.971
Systolic PAP (mmHg)		34.04 ± 10.26	35 ± 12.13	0.638
LVEF (%)	> 50	154 (75.5%)	4 (36.4%)	0.009^∗^
	=< 50	50 (24.5%)	7 (63.6%)	

LV enlargement	No	190 (93%)	11 (91.7%)	0.588
	Yes	14 (6.9%)	1 (8.3%)	

LA enlargement	No	65 (30.9%)	6 (50%)	0.215
	Yes	139 (68.1%)	6 (50%)	

PH	No	135 (66.2%)	7 (58.3%)	0.550
	Yes	69 (33.8%)	5 (41.7%)	

Kind of HCM	Obstructive	80 (39.2%)	2 (16.7%)	0.138
	Nonobstructive	124 (60.8%)	10 (83.3%)	

RVH	No	188 (92.2%)	10 (83.3%)	0.605
	Yes	16 (7.8%)	2 (16.7%)	

SAM of AMVL	No	83 (40.7%)	9 (75%)	0.019
	Yes	121 (59.3%)	3 (25%)	

Significant LVOT gradient at rest	No	142 (69.6%)	11 (91.7%)	0.187
	Yes	62 (30.4%)	1 (8.3%)	

Significant provoked LVOT gradient	No	108 (73.5%)	8 (80%)	0.734
	Yes	39 (26.5%)	2 (20%)	

*Note:* LVEDVi: left ventricular diastolic volume index, LVEDD: left ventricular diastolic diameter, HTN: hypertension, HCM: hypertrophic cardiomyopathy.

Abbreviations: AF, atrial fibrillation; AI, aortic insufficiency; AMVL, anterior mitral valve leaflet; BSA, body surface area; DBP, diastolic blood pressure; DM, diabetes mellitus; ICD, implanted cardioverter defibrillator; LA, left atrium; LAVi, left atrium volume index; LV, left ventricle; LVEF, left ventricle ejection fraction; LVESD, left ventricular end‐systolic diameter; LVOT, left ventricular outflow tract; MR, mitral regurgitation; PAP, pulmonary arterial pressure; PH, pulmonary hypertension; RVH, right ventricle hypertrophy; SAM, systolic anterior motion; SBP, systolic blood pressure; SOV, sinus of Valsalva.

^∗^ *p* value < 0.05.

According to echocardiographic data, among 216 patients, 12 patients (5.6%) had isolated SOV dilation, 56 patients (26.3%) had isolated ascending aorta dilation, and 8 patients (3.7%) had concurrent dilation of both SOV and ascending aorta.

The analysis of the prevalence of dilated ascending aorta in relation to the characteristics and past medical history indicated that age (*p* value = 0.001), HTN (*p* value = 0.001), and BSA (*p* value = 0.002) had significant correlation with dilated ascending aorta. Age had a more pronounced correlation with dilated ascending aorta, as the average age of patients with dilated ascending aorta was 56.8 ± 13.93 years, greatly exceeding that of patients without this condition. There was no significant correlation between dilated ascending aorta and sex (*p* value = 0.641).

Among echocardiographic data, only LVESD had a significant correlation with dilated ascending aorta (*p* value = 0.002). There were no significant differences in the degree of MR and AI between patients with and without dilated ascending aorta (*p* value = 0.54 and 0.15, respectively).

According to MRI, myocardial fibrosis was evaluated in patients with and without ascending aorta dilation. The prevalence of myocardial fibrosis was higher in patients with dilated ascending aorta compared to those with normal size (89.5% vs. 67.6%), but this difference did not reach statistical significance (*p* value = 0.06).

Based on the variables evaluated, the prevalence of dilated SOV showed significant correlation with age (*p* value = 0.001), that patients with dilated SOV were older than those without dilated SOV. Also, there was a significant correlation between dilated SOV and HTN (*p* value = 0.013) and BSA (*p* value = 0.004).

Evaluating echocardiographic data showed a significant negative correlation between dilated SOV and LVEF (*p* value = 0.009) and also a positive correlation between dilated SOV and the presence of AI (*p* value = 0.008). There were no significant differences in the degree of MR between patients with and without dilated SOV (*p* value = 0.73). Of the 87 patients that underwent cardiac MRI, there was no significant correlation between the presence of myocardial fibrosis and dilated SOV (*p* value = 0.316).

Table 4 shows a logistic regression model fit for dilated ascending aorta. The results indicate that, among the variables of interest, only weight, age, LVESD, HTN, PH, and RVH had a significant relationship with dilated ascending aorta, while the other variables did not show a statistically significant association: For each additional year of age, the odds of dilated ascending aorta increase by 6% (OR = 1.063, CI 95% = 1.022–1.150). For the variable LVESD, for every unit increase in LVESD, the odds of dilated ascending aorta increase by 25% (OR = 3.250, CI 95% = 1.155–9.150). Additionally, for every unit decrease in weight, the odds of dilated ascending aorta decrease by 1.05 times (OR = 0.949, CI 95% = 0.910–0.989). Individuals with PH have 14.7 times lower odds of having dilated ascending aorta compared to those without PH (OR = 7.14, CI 95% = 0.025–0.779). Patients with HTN have a 5 times higher odds of having dilated ascending aorta compared to those without HTN (OR = 5.49, CI 95% = 1.583–19.084), and individuals with RVH have a 4.9 times higher odds of having dilated ascending aorta compared to those without RVH (OR = 4.99, CI 95% = 1.078–23.173).

**TABLE 4:** Results of the logistic regression model fit for dilated ascending aorta.

Variables		B	S.E.	Wald	*p* value	Exp (B)	95% CI for EXP (B)	
							Lower	Upper
Age		0.061	0.020	9.279	0.002^∗^	1.063	1.022	1.105

Height		−0.033	0.030	1.195	0.274	0.967	0.911	1.027

Weight		−0.053	0.021	6.130	0.013^∗^	0.949	0.910	0.989

Heart rate		0.015	0.047	0.106	0.745	1.015	0.926	1.114

SBP		0.021	0.028	0.544	0.461	1.021	0.966	1.079

DBP		−0.060	0.047	1.595	0.207	0.942	0.858	1.034

AF	Yes	0.226	0.683	0.109	0.741	1.253	0.328	4.782
	No	The basis considered—reference						

LVEDD		−0.501	0.564	0.791	0.374	0.606	0.201	1.828

LVEDVI		0.006	0.021	0.095	0.758	1.006	0.966	1.049

LVESD		1.179	0.528	4.982	0.026^∗^	3.250	1.155	9.150

LAVI		−0.011	0.013	0.736	0.391	0.989	0.965	1.014

SAM	Yes	0.267	0.522	0.261	0.609	1.306	0.469	3.633
	No	The basis considered—reference						

PAP		0.051	0.040	1.636	0.201	1.053	0.973	1.139

LA enlargement	Yes	0.133	0.610	0.048	0.827	1.142	0.345	3.779
	No	The basis considered—reference						

Al	Yes	0.190	0.511	0.138	0.710	1.209	0.444	3.293
	No	The basis considered—reference						

PH	Yes	−1.969	0.877	5.042	0.025^∗^	0.140	0.025	0.779
	No	The basis considered—reference						

HTN	Yes	1.704	0.635	7.202	0.007^∗^	5.497	1.583	19.084
	No	The basis considered—reference						

DM	Yes	−1.642	0.974	2.840	0.092	0.194	0.029	1.307
	No	The basis considered—reference						

ICD	Yes	0.733	0.573	1.637	0.201	2.081	0.677	6.394
	No	The basis considered—reference						

RVH	Yes	1.609	0.783	4.229	0.040^∗^	4.999	1.078	23.174
	No	The basis considered—reference						

LVEF	< 50	0.469	0.664	0.499	0.480	1.599	0.435	5.879
	> 50	The basis considered—reference						

LV enlargement	Yes	0.006	1.034	0.000	0.995	1.006	0.133	7.638
	No	The basis considered—reference						

^∗^ *p* value < 0.05.

Table 5 shows a logistic regression model fit for dilated SOV. The results revealed that, among the variables of interest, only weight and age had a significant relationship with dilated SOV, while the other variables did not show a statistically significant association. For each additional year of age, the odds of dilated SOV increase by 1.11 times (OR = 1.117, CI 95% = 1.034–1.207). Additionally, for every unit decrease in weight, the odds of dilated SOV decrease by 1.092 times (OR = 0.915, CI 95% = 0.837–0.999).

**TABLE 5:** Results of the logistic regression model fit for dilated SOV.

Variables		B	S.E.	Wald	*p* value	Exp (B)	95% C.I. for EXP (B)	
							Lower	Upper
Age		0.111	0.040	7.852	0.005^∗^	1.117	1.034	1.207

Height		0.008	0.050	0.024	0.878	1.008	0.914	1.112

Weight		−0.089	0.045	3.935	0.047^∗^	0.915	0.837	0.999

SBP		−0.036	0.051	0.495	0.482	0.965	0.873	1.066

DBP		0.089	0.076	1.359	0.244	1.093	0.941	1.268

AF	Yes	−1.092	1.257	0.755	0.385	0.336	0.029	3.938
	No	The basis considered—reference						

SAM	Yes	−0.703	0.863	0.664	0.415	0.495	0.091	2.685
	No	The basis considered—reference						

LVEDVI		0.017	0.045	0.134	0.714	1.017	0.931	1.111

LVEDD		−0.803	0.676	1.410	0.235	0.448	0.119	1.686

LAVI		−0.040	0.032	1.574	0.210	0.961	0.902	1.023

^∗^ *p* value < 0.05.

## 4. Discussion

In our tertiary center of cardiovascular disease in Iran, we evaluated the prevalence of aortic dilation in patients with HCM, and also investigated the related factors [16]. We analyzed all data with indexed values of SOV and ascending aorta that were adjusted with BSA and gender.

In our study, 5.6% of enrolled patients with HCM had dilated SOV, 26.3% had dilated ascending aorta, and 3.7% had concurrent dilation of both SOV and ascending aorta. Our study showed that the prevalence of dilated ascending aorta is higher than that of dilated SOV in HCM patients. The prevalence of dilated ascending aorta size was much greater in our study compared with the largest study about the aorta size in patients with HCM [17], which reported 13%. This difference may be due to genetic factors in Iran, and also that our values were not adjusted by age, as in their study, when the aorta sizes were not adjusted by sex and age, the prevalence was 18%. But our findings are consistent with some cohorts, where the prevalence of aortic dilation in HCM varied, reaching up to 29% in some cohorts [17, 18]. Although HCM is traditionally viewed as a myocardial disease, this study supports the growing body of evidence indicating that aortic dilation may represent an important yet under‐recognized component of the disease [19, 20].

Our results showed that advanced age was correlated significantly with dilated ascending aorta and dilated SOV, as patients with dilated ascending aorta had a mean age of 56.80 years, whereas those with dilated SOV had a mean age of 63.08 years. The rise in age may correlate with structural alterations and diminished flexibility of the aorta wall that happened throughout time. Prior research has substantiated this correlation, indicating that diminished aortic elasticity and increased wall stiffness correlate with an elevated risk of aortic dilatation [17–19, 21]. So, in older patients with HCM, closer follow‐up for aorta size may be needed.

This study showed that hypertension and BSA are significant predictors of both ascending aorta and SOV dilation. These associations are biologically plausible, as hypertension is known to contribute to aortic wall degeneration and increased aortic stiffness [6, 17]. Hypertension can result in the progressive deterioration of elastin and collagen due to increased mechanical stress on the aorta wall, ultimately resulting in aortic dilation [19, 20]. Previous studies have similarly demonstrated that systemic hypertension in HCM patients amplifies the mechanical stress on the aortic wall, predisposing to progressive dilation [17, 22].

The association between higher BSA and aortic dilation is important because indexed values for BSA were used in our study. Our study did not find significant sex differences in the prevalence of aortic dilation, which contrasts with some reports indicating male predominance in aortic pathology but aligns with the notion that sex differences may be less pronounced in the HCM population in Iran.

Interestingly, echocardiographic parameters such as maximum LV wall thickness and LVOT obstruction were not significantly associated with aortic dilation in this study. This finding may challenge earlier assumptions that hemodynamic forces from LVOT obstruction are the primary drivers of aortopathy in HCM [4, 20]. It instead highlights the possible role of intrinsic aortic wall abnormalities or coexisting genetic factors unrelated to sarcomere mutations [5, 19]. However, we found that LVESD was significantly associated with ascending aorta dilation and was higher in this group. This may indicate a relationship between ventricular remodeling and the mechanical load on the proximal aorta. RVH and PH also emerged as significant correlates in multivariate analysis, underscoring the possible contribution of broader biventricular and vascular remodeling in these patients. PH appeared inversely related to aortic dilation. This inverse relationship may be attributable to reduced left ventricular forward stroke volume and diminished pulsatile stress on the ascending aorta in the setting of advanced right‐sided pressure overload.

The significant negative association between SOV dilation and LVEF = < 50% that defined as burned out HCM, may indicate that patients with advanced myocardial disease have the same underlying factors that lead to aortic dilation.

The association of dilated SOV with AI also underscores the clinical relevance of aortic root dilation, as AI may both contribute to and result from altered aortic root geometry. It is worth noting that we excluded patients with moderate AI or greater.

The higher prevalence of myocardial fibrosis in patients with dilated ascending aorta observed in cardiac MRI, though not statistically significant, hints at a possible link between myocardial structural changes and aortic pathology. This trend suggests that patients with more advanced myocardial disease might be related to the same factors that lead to aortic dilation, including similar pathological mechanisms such as extracellular matrix remodeling or underlying genetic predisposition, and contributing factors such as age and HTN that promote both aortic wall and myocardial structural changes, although larger studies are needed to confirm this relationship. As in our study, only 87 patients had an MRI, so this can limit our results, and maybe if there were more cases, the result became significant.

This study’s findings highlight that factors including age, HTN, and particular anatomical measurements serve as critical indicators for identifying at‐risk patients that can guide screening programs and preventive measures. The awareness to detect early alterations in aortic dimensions may enhance care of these patients [17, 18].

For exploratory follow‐up assessment, available medical records were reviewed for up to 1 year after the index echocardiographic evaluation whenever subsequent clinical data were accessible. Follow‐up information included repeat echocardiographic findings, major arrhythmic events, ICD interventions, aortic dissection, major aortic complications, and mortality. During the 1‐year follow‐up period, no cases of aortic dissection, major aortic complications, aortic surgery, or mortality were identified. Due to the retrospective nature of the study, follow‐up data were not uniformly available for all patients and were therefore not included in the primary statistical analyses.

### 4.1. Limitations

This study presented certain limitations that must be taken into account when analyzing the results. The retrospective design of the study and single‐center setting may have introduced bias in data gathering. Furthermore, due to the retrospective nature of the study, complete follow‐up data were not available for all patients, limiting precise assessment of longitudinal changes in ascending aortic dimensions progression and related clinical outcomes. The insufficient examination of genetic characteristics linked to these disorders may represent a significant constraint necessitating additional research. The relatively small number of patients with cardiac MRI limits the power to detect significant relationships involving myocardial fibrosis.

## 5. Conclusion

Aortic dilation is a relatively common finding in patients with HCM and is significantly associated with age, HTN, and structural cardiac remodeling. Routine evaluation of the ascending aorta and SOV in HCM patients may aid early diagnosis and management of this potential complication. Our single‐center findings support previous reports of an association between aortic dilatation and HCM, but confirmation in larger, multicenter studies is warranted before influencing clinical guidelines.

## Funding

No funding was received for this manuscript.

## Ethics Statement

The ethical code is IR.IUMS.FMD.REC.1401.464.

## Conflicts of Interest

The authors declare no conflicts of interest.

## Data Availability

The data that support the findings of this study are available from the corresponding author upon reasonable request.
